# Supplemental oxygen users with pulmonary fibrosis perceive greater dyspnea than oxygen non-users

**DOI:** 10.1186/s40248-015-0035-y

**Published:** 2015-11-30

**Authors:** Mengshu Cao, Frederick S. Wamboldt, Kevin K. Brown, Jonathon Hickman, Amy L. Olson, Joshua J. Solomon, Jeffrey J. Swigris

**Affiliations:** Department of Respiratory Medicine, Nanjing Drum Tower Hospital, the affiliated Nanjing University Medical School, 321 Zhongshan Road, Nanjing, Jiangsu 210008 China; Division of Pulmonary, Critical Care, Sleep and Behavioral Medicine, Department of Medicine, National Jewish Health, Denver, Colorado USA; Interstitial Lung Disease Program, Department of Medicine, National Jewish Health, 1400 Jackson Street, Denver, 80206 Colorado USA; Department of Internal Medicine, Saint Joseph Hospital, 1375 E 19th Ave, Denver, 80218 Colorado USA

**Keywords:** Interstitial lung disease, Pulmonary fibrosis, Dyspnea, Supplemental oxygen, 6-min walk test

## Abstract

**Background:**

Exertional dyspnea is a hallmark symptom of fibrosing interstitial lung disease (fILD), and oxygen (O_2_) desaturation is common among patients with fILD. Supplemental O_2_ is prescribed to maintain normoxia and alleviate dyspnea. We sought to better understand the associations between O_2_ and dyspnea in fILD during the 6-min walk test (6MWT).

**Methods:**

1326 fILD patients compose the sample group. Borg dyspnea and other 6MWT variables were compared between subjects who performed the test without (non-users) versus with O_2_ (users).

**Results:**

There were 812 users and 514 non-users; users were older, more likely to have smoked, had greater body mass index, and had more severe fILD. Despite a similar 6-min SpO_2_, users perceived greater dyspnea than non-users (Borg 3.9 ± 2.0 vs 2.9 ± 1.7, *p* < 0.0001). Whether subjects became hypoxemic (6-min SpO_2_ < 89 %) or not during the walk, the results were the same: users perceived greater dyspnea than non-users (hypoxemic: users 3.5 ± 2.1 vs non-users 2.7 ± 1.8, *p* < 0.0001; non-hypoxemic: users 3.4 ± 1.9 vs non-users 2.4 ± 1.6, *p* < 0.0001). Among subjects who did not desaturate (SpO_2_ drop < 4 %), users walked a shorter distance (944.9 ± 367.0 vs 1385.3 ± 322.4 feet, *p* < 0.0001) but perceived greater dyspnea than non-users (3.3 ± 1.6 vs 2.3 ± 1.7, *p* = 0.005). No combination of potentially influential predictor variables entered in multivariate models explained more than 11 % of the variance in dyspnea ratings.

**Conclusion:**

Dyspnea is a complex perception, and in patients with fILD, O_2_ may lessen, but does not resolve, it. Further research is needed to clarify why fILD patients who use O_2_ perceive greater levels of dyspnea with activity than O_2_ non-users.

## Background

The interstitial lung diseases (ILD) comprise several diffuse parenchymal lung diseases whose causes are unknown or include exposures (e.g., dust, drug, aerosolized organic antigen) or underlying connective tissue disease (CTD). Regardless of cause, fibrotic ILD (fILD) is typically progressive and incurable. Exertional dyspnea, the hallmark symptom of fILD, impairs physical functioning and quality of life (QOL) and is often associated with peripheral oxygen desaturation (SpO_2_).

The six-minute walk test (6MWT) is commonly used as a measure of submaximal exercise capacity in patients with fILD. Along with distance walked (6MWD), SpO_2_, heart rate and dyspnea ratings are often collected as part of the 6MWT and used to assess disease status. Dyspnea—the perception of “breathing discomfort”—is due to a number of complex physical, psychological, social, environmental and interwoven physiological factors [1]. In fILD, dyspnea is due to reduced lung compliance, inability to expand tidal volume in response to respiratory drive, as well as the elevated work and oxygen cost of breathing [2]. Although dyspnea is a personalized perception, it is experienced and described similarly among patients with the same respiratory disease. For example, during symptom-limited incremental cycle exercise tests, ‘unsatisfied inspiratory effort’ and ‘rapid breathing’ are used to describe dyspnea by patients with fILD—but not by healthy controls [3]. Investigators have observed that although patients with fILD desaturated to a greater degree than patients with chronic obstructive pulmonary disease (COPD), patients with COPD perceived greater dyspnea. In that study, SpO_2_ was an independent predictor of dyspnea severity in patients with fILD but not in those with COPD. Among patients with fILD, SpO_2_ explained only a quarter of the variance in dyspnea ratings [4].

Although supplemental oxygen (O_2_) is commonly prescribed to patients with fILD to maintain normoxia, in hopes of relieving dyspnea (and by extension, improving physical functioning and QOL), few studies have aimed to decipher the beneficial effects of O2 in these patients [5, 6]. Through this study, we sought to examine how dyspnea ratings from patients who use O_2_ compare with those from patients who do not use O_2_.

## Methods

### Study subjects

The study  group was composed of 1326 patients with fILD evaluated at National Jewish Health (NJH) from January 1, 2008 to December 30, 2014. We formed the cohort by querying the NJH research database for patients with fILD who completed at least one 6MWT. Patients with underlying connective tissue disease (CTD) were excluded; thus, the overwhelming majority of subjects had idiopathic pulmonary fibrosis (IPF), idiopathic nonspecific interstitial pneumonia (iNSIP) or chronic hypersensitivity pneumonia (cHP), with diagnoses made in accordance with accepted criteria [7–10]. The study was approved by the NJH Institutional Review Board (IRB; study #2868) which waived the requirement for written, informed consent.

### 6MWT

The 6MWT was conducted similarly in all patients (whether users or non-users), by trained technicians at NJH, according to published guidelines with slight modification [11]. Per standard operating procedure at NJH, the 6MWT is terminated if SpO_2_ drops below 80 %. To maintain reliability in the 6MWT outcome of most interest (distance), we  tried to hold constant as many other variables as possible. Thus, a patient  performed all 6MWT on the same O_2_ l flow, unless or until he  was unable to walk for a full six minutes without SpO_2_ falling below 80 %. We included in our analyses data only from patients who walked for a full six minutes. For patients who completed multiple 6MWT, we selected the first test. Ratings for dyspnea and exertion were assessed immediately after completion of the test by the technician and using the CR10 Borg scale (range 0–10, with higher scores connoting greater dyspnea or exertion as appropriate) [12]. The minimal clinically important difference for the Borg scale is reported to be one point [13].

### Statistical analysis

Summary statistics were generated for baseline data with the sample stratified on whether O_2_ was used (users) or not (non-users) during the 6MWT. Student’s *t*-tests were used for between-groups comparisons of continuous variables. Cochran-Mantel-Haenszel, Chi square or Fisher’s exact tests were used as appropriate for between-groups comparisons of categorical variables. We used Pearson correlation coefficients to express associations between dyspnea ratings and other variables. We used linear regression to examine associations between dyspnea ratings and other variables while controlling for potentially influential predictors. We considered *p* < 0.05 to represent statistical significance. Analyses were performed using SAS version 9.3 statistical software (SAS, Inc.; Cary, NC).

## Results

The study group comprised 812 users and 514 non-users. On average, users were older, had greater impairments in pulmonary physiology and had shorter 6MWD than non-users. Despite a similar SpO_2_ at six minutes (88.1 % vs. 88.7 %), users perceived significantly greater dyspnea than non-users (Table 1).

**Table 1:** Baseline characteristics for O2 users and O2 non-users with fILDs

	O2 (*N* = 812)	No O2 (*N* = 514)	*P*-
Age (years)	68.3 ± 10.5	66.6 ± 11.0	0.004
Female (%)	327 (40.3)	211 (41.1)	0.78
Smoking*	4 (0.6 %)	3 (0.6 %)	0.003
Present	471 (65.9 %)	277 (57.4 %)	
Past	240 (33.6 %)	203 (42.0 %)	
Never			
BMI	30.3 ± 6.9	28.5 ± 5.5	<0.0001
IPF diagnosis (%)	303 (37.3)	170 (33.1)	0.12
FVC% within 30 days**	61.3 ± 18.9	77.5 ± 17.4	<0.0001
DLCO% within 30 days***	37.5 ± 12.8	56.1 ± 16.5	<0.0001
6MWD (feet)	1070.6 ± 361.4	1421.4 ± 343.7	<0.0001
Borg Dyspnea	3.9 ± 2.0	2.9 ± 1.7	<0.0001
Borg Exertion****	3.2 ± 2.1	2.4 ± 1.8	<0.0001
HR baseline	79.7 ± 13.9	78.1 ± 13.2	0.03
HR at 6 min	109.7 ± 15.9	110.9 ± 15.8	0.18
HR rise	30.0 ± 14.5	32.8 ± 13.7	0.0004
SpO2 baseline	97.4 ± 1.9	95.2 ± 1.7	<0.0001
SpO2 at 6 min	88.1 ± 5.3	88.7 ± 5.4	0.04
SpO2 drop	9.2 ± 5.5	6.4 ± 5.2	<0.0001

Values are mean and standard deviation or count (percent); *IPF* idiopathic pulmonary fibrosis; *BMI* body mass index; *FVC%* percent predicted forced vital capacity; *DLCO%* percent predicted diffusing capacity of the lung for carbon monoxide; *6MWD* distance walked during six-minute walk test (6MWT); *SpO2* peripheral oxygen saturation; *HR* heart rate; *O2* completed 6MWT using supplemental oxygen; *No O2* completed 6MWT without using supplemental oxygen; *N = 715 for O2 users and 483 for non-users; **N = 446 for O2 users and 251 for non-users; ***N = 211 for O2 users and 123 for non-users; ****N = 672 for O2 users and 415 for non-users

In both users and non-users, dyspnea was correlated with certain other variables; however, all correlations were weak (Table 2). In both subgroups, dyspnea was inversely correlated with 6MWD. Among the 791 subjects whose SpO_2_ fell below 89 %, there were nearly twice as many users as non-users (Table 3). Although the SpO_2_ at six minutes was similar (85.3 % vs. 86.0 %), dyspnea ratings among users were significantly higher than in non-users. The same was true for subjects whose SpO_2_ remained 89 % or greater for the duration of the 6MWT: despite identical mean SpO_2_ values at six minutes (91.4 % vs. 91.4 %), dyspnea ratings were significantly higher among users than in non-users (Table 4).

**Table 2:** Correlation between dyspnea rating and other variables for O2 users and O2 non-users

	O2 (*N* = 812)	No O2 (*N* = 514)
6MWD	−0.28	−016
	<0.0001	0.0003
BMI	0.15	0.14
	<0.0001	0.002
HR baseline	0.07	0.03
	0.04	0.53
HR at 6 min	0.08	0.16
	0.02	0.0003
HR rise	0.02	0.15
	0.49	0.0003
SpO2 baseline	0.03	−0.14
	0.47	0.002
SpO2 at 6 min	−0.19	−0.21
	<0.0001	<0.0001
SpO2 drop	−0.19	−0.17
	<0.0001	<0.0001

Values are correlation coefficient (top) and p value (bottom); *BMI* body mass index; *6MWD* distance walked during six-minute walk test (6MWT); *HR* heart rate; *SpO*2 peripheral oxygen saturation; *O2* completed 6MWT using supplemental oxygen; *6MWD* distance walked during six-minute walk test (6MWT); *O2* completed 6MWT using supplemental oxygen; No *O2* completed 6MWT without using supplemental oxygen

**Table 3:** Dyspnea and other results for O2 users and O2 non-users among subjects whose nadir SpO2 was < 89 %

	O2 (*N* = 439)	No O2 (*N* = 252)	*P*
6MWD (feet)	1051 ± 382.7	1415.1 ± 370.5	<0.0001
BMI	30.2 ± 6.8	28.8 ± 5.2	0.003
Borg Dyspnea	4.3 ± 2.0	3.4 ± 1.7	<0.0001
Borg Exertion*	3.5 ± 2.1	2.7 ± 1.8	<0.0001
HR baseline	80.4 ± 13.7	78.2 ± 13.3	0.04
HR at 6 min	112.3 ± 16.0	112.9 ± 16.2	0.62
HR rise	31.9 ± 14.8	34.7 ± 15.3	0.02
SpO2 baseline	97.2 ± 2.0	94.6 ± 1.7	<0.0001
SpO2 at 6 min	85.3 ± 3.5	86.0 ± 3.2	0.02
SpO2 drop	11.8 ± 4.1	8.6 ± 3.3	<0.0001

Values are mean and standard deviation; *6MWD* distance walked during six-minute walk test (6MWT), *O2* completed 6MWT using supplemental oxygen, *No O2* completed 6MWT without using supplemental oxygen, *SpO2* peripheral oxygen saturation; *N = 355 for O2 users and 204 for non-users

**Table 4:** Dyspnea and other results for O2 users and O non-users among subjects whose nadir SpO2 was 89 % or greater (nadir SpO2 ≥ 89 %)

	O2 (*N* = 373)	No O2 (*N* = 262)	*P*
6MWD	1092.4 ± 333.8	1427.4 ± 316.4	<0.0001
BMI	30.4 ± 6.9	28.2 ± 5.8	<0.0001
Borg Dyspnea	3.4 ± 1.9	2.4 ± 1.6	<0.0001
Borg Exertion*	2.9 ± 2.1	2.2 ± 1.7	<0.0001
HR baseline	79.0 ± 14.0	78.0 ± 13.0	0.37
HR at 6 min	106.7 ± 15.3	109.0 ± 15.1	0.06
HR rise	27.7 ± 13.7	31.0 ± 11.8	0.001
SpO2 baseline	97.6 ± 1.7	95.7 ± 1.6	<0.0001
SpO2 at 6 min	91.4 ± 5.2	91.4 ± 5.7	0.97
SpO2 drop	6.5 ± 2.4	4.6 ± 1.9	<0.0001

Values are mean and standard deviation; *6MWD* distance walked during six-minute walk test (6MWT), *O2* completed 6MWT using supplementaloxygen, *No O2* completed 6MWT without using supplemental oxygen,*HR* heart rate, *SpO2* peripheral oxygen saturation; *N = 317 for O2 users and 211 for non-users

Results were similar for the 883 subjects (572 users and 311 non-users) with a history of smoking: the SpO_2_ values at six minutes were similar (87.9 % vs. 88.4 %), and dyspnea ratings were higher among users than in non-users (3.8 ± 2.0 vs. 2.9 ± 1.7, *p* < 0.0001). For the 473 subjects (303 users and 170 non-users) with IPF, the SpO_2_ values at six minutes were the same (86.7 % vs. 86.9 %), and dyspnea ratings were higher among users than in non-users (3.8 ± 2.1 vs. 2.9 ± 1.6, *p* < 0.0001). Among the 118 subjects whose SpO_2_ never dropped by more than three points from baseline (rest), although minute-six SpO_2_ was higher in users than in non-users, dyspnea ratings among users were significantly higher than in non-users (Table 5).

**Table 5:** Dyspnea and other results for O2 users and O2 non-users among subjects whose SpO2 fell by <4 % during the walk

	O2 (*N* = 36)	No O2 (*N* = 82)	*P*
6MWD (feet)	944.9 ± 367.0	1385.3 ± 322.4	<0.0001
BMI	31.6 ± 6.8	27.6 ± 5.3	0.001
Borg Dyspnea	3.3 ± 1.6	2.3 ± 1.7	0.005
Borg Exertion*	2.7 ± 2.0	2.2 ± 1.7	0.20
HR baseline	81.3 ± 15.3	79.9 ± 14.7	0.64
HR at 6 min	99.6 ± 11.7	107.6 ± 15.6	0.007
HR rise	18.2 ± 16.1	27.6 ± 10.5	0.002
SpO2 baseline	96.6 ± 2.5	95.2 ± 1.9	0.0009
SpO2 at 6 min	95.2 ± 2.3	93.4 ± 2.1	<0.0001
SpO2 drop	1.1 ± 3.1	1.8 ± 1.0	0.18

Values are mean and standard deviation; *6MWD* distance walked during six-minute walk test (6MWT), *O2* completed 6MWT using supplemental oxygen, *No O2* completed 6MWT without using supplemental oxygen, *HR* heart rate, *SpO2* peripheral oxygen saturation; *N = 30 for O2 users and 67 for non-users

Results from the linear regression analysis are presented in Table 6. While controlling for various combinations of predictors, O_2_ use remained a significant predictor of dyspnea rating. As revealed by the R-squared values, none of the combinations of variables explained more than minimal variance in dyspnea ratings.

**Table 6:** Linear regression models showing association between dyspnea ratings and other variables

	Model 1	Model 2	Model 3	Model 4	Model 5
Intercept	2.50 ± 0.10	3.31 ± 0.26	3.24 ± 0.27	5.37 ± 0.31	1.96 ± 0.36
	<0.0001	<0.0001	<0.0001	<0.0001	<0.0001
Used O2	0.77 ± 0.11	0.66 ± 0.12	0.68 ± 0.12	0.42 ± 0.13	0.54 ± 0.12
	<0.0001	<0.0001	<0.0001	0.001	<0.0001
BMI					0.05 ± 0.01
					<0.0001
SpO2 drop	0.07 ± 0.01	0.06 ± 0.01	0.06 ± 0.01		0.06 ± 0.01
	<0.0001	<0.0001	<0.0001		<0.0001
FVC%		−0.01 ± 0.003	−0.01 ± 0.003	−0.01 ± 0.0002	−0.01 ± 0.002
		0.001	0.001	0.008	<0.0001
6MWD				−0.001 ± 0.002	
				<0.0001	
HR rise			0.003 ± 0.004		
			0.43		
R-square	0.09	0.09	0.09	0.11	0.11

Values are coefficients and standard error (top) and *p* value (bottom); *6MWD* distance walked during six-minute walk test (6MWT), *O2* completed 6MWT using supplemental oxygen, No *O2* completed 6MWT without using supplemental oxygen, *HR* heart rate, *SpO2* peripheral oxygen saturation

## Discussion

We examined patients with fILD and found that those who used O_2_ during a 6MWT consistently experienced more severe dyspnea than those who did not use O_2_. Data on the effects of O_2_ in patients with fILD are surprisingly limited, and much of the information on the potential benefits of O_2_ that is used in clinical decision-making with fILD patients, is based solely on scientific rationale or borrowed from the COPD literature. In two Letters to the Editor, investigators described the results of retrospective studies in which they examined the within-subject beneficial effects of O_2_ on various outcome measures collected around 6MWTs [5, 6]. In one study, investigators observed that, in 52 patients with fILD, during a second 6MWT for which O_2_ was administered according to a semi-quantitative algorithm aimed at maintaining SpO_2_ at (closer-to) acceptable levels, distance walked, nadir SpO_2_ and Borg score improved (by one point) over values obtained during a baseline 6MWT. The results were similar for the subgroup of subjects with either idiopathic pulmonary fibrosis (IPF) or NSIP, in whom O_2_ administration resulted in improved dyspnea (median Borg scores dropped from 4.25 to 3.25) and in the nadir SpO_2_ increasing from 75 to 83 %. In the other study of 70 subjects (all with IPF), Frank and her colleagues found that, compared to a baseline 6MWT performed on ambient air or with inadequate O_2_ flow, administering O_2_ led to increased distance walked and improved nadir SpO_2_, but dyspnea did not change (mean Borg score 4.8 ± 2.1 vs. 4.5 ± 2.2) [5].

Dyspnea is a complex perception that depends on the integration of multiple inputs from several sources. Blood oxygen level is but one of those sources, and the weak correlation we observed between dyspnea rating and minute-six SpO_2_ affirms it is far from the main contributor. Other contributors include neural inputs arising from receptors in the airways and lung parenchyma, peripheral locomotor and respiratory muscles, and central and peripheral chemoreceptors, along with corollary neuronal discharge arising from the brainstem and cortical motor centers [14].

In our study, patients who used O_2_ consistently started with a higher SpO_2_ than non-users, and although SpO_2_ declined to a greater degree during the walk in users than non-users (9 % vs. 6 %), minute-six SpO_2_ was the same in both groups (88 %). Perhaps has dyspnea more to do with SpO_2_ decline from baseline than the absolute SpO_2_ at the time of dyspnea rating? Our results suggest this is not the case: the correlation between dyspnea and SpO_2_ decline was the same (weak) as the correlation between dyspnea and minute-six SpO_2_. However, in the subgroup of patients who did not desaturate at all during the walk (SpO_2_ decline < 4 points), despite O_2_ users having a higher minute-six SpO_2_ than non-users (95 % vs. 93 %), O_2_ users perceived greater dyspnea (mean Borg scores 3.4 vs. 2.4).

In our statistical models controlling for either minute-six (data not shown) or decline-from-baseline in SpO_2_ (i.e., SpO_2_ drop), each of these SpO_2_ measures was a significant predictor of dyspnea, and in each model, O_2_ use remained an independent predictor of dyspnea. However, each model explained minimal variance in dyspnea scores—again, confirming that dyspnea relies on more inputs than simply blood oxygen.

We suspect users in our study perceived greater dyspnea intensity than non-users because of a complex interaction of elements, including those related to conduct of the 6MWT and perhaps certain neurophysiological factors. At our center, in an attempt to maintain reliability in the 6MWT outcome of most interest (distance), we try to hold constant as many other variables as possible. Thus, a patient performs all 6MWT on the same O_2_ l flow, unless or until he is unable to walk for a full six minutes (in which case flow is reset for subsequent 6MWTs). Because of this practice, on certain occasions, patients at our center may perform their 6MWT on O_2_ l flows below what they use with exertion at home. In our study, this “intentional under-dosing” of O_2_ flow—to maintain reliability—could have driven dyspnea ratings up in O_2_ users. Unfortunately, with this data set, we are unable to determine when this under-dosing might have occurred. Regardless, this “intentional under-dosing” explanation would seem not to apply to subjects whose O_2_ was dosed adequately enough to maintain an acceptable SpO_2_ throughout the test, including the over 600-patient subgroup whose SpO_2_ remained above 88 %, or the greater than 100-patient subgroup whose saturations did not decline at all. In both these subgroups, O_2_ users rated their dyspnea as more intense than non-users.

Also at our center, patients who use O_2_ at home either carry or pull their O_2_ delivery device while completing their 6MWT. Having to move this excess weight over distance—or altered chest wall mechanics resulting from carrying or pulling the device—could add to dyspnea. To our knowledge, this has yet to be examined, but we believe it  deserves investigation. If carrying or pulling the delivery device is found to add significantly to dyspnea intensity, this could be a target for therapeutic intervention.

Another alternative explanation is that dyspnea truly depends greatly on arterial oxygen but SpO_2_ was an especially inaccurate reflection of it in this cohort; we doubt this was the case, but if it were, we would expect the inaccuracies to affect both users and non-users equally. We excluded patients with underlying CTD in the hopes of limiting the influence of vascular abnormalities like Raynaud’s or pulmonary hypertension.

Various physical factors unrelated to the lungs, SpO_2_ or other aspects of oxygen delivery also must be considered as potential explanations for our results. While exerting (and into recovery), subnormal lung compliance in patients with ILD induces rapid-shallow breathing. The physical sensation—and mental/emotional impact—of this breathing pattern, which occurs to some degree when patients with ILD exert to any degree, is expected to influence dyspnea ratings. How subjects internally considered, weighed and integrated each component (physical or mental/emotional) as they made their ratings for “breathlessness” is unknown. Compared with non-users, O_2_ users had lower FVC% and, by deduction, lower lung compliance than non-users—a factor expected to hasten and heighten rapid-shallow breathing. We do not measure respiratory rate during the 6MWT at our center, so we are unable to determine whether O_2_ users had higher respiratory rates than non-users. Additional studies aimed at discerning whether or how much respiratory rate (and other physical or emotional components) contributes to exertional dyspnea ratings are needed. Although we are unable to comment on directly-observed respiratory rate, FVC% as a marker of lung compliance could be considered a reasonable surrogate for respiratory rate. In statistical models that included O_2_ use, and controlled for FVC%, O_2_ use remained a significant predictor of dyspnea. In a comprehensive appraisal of dyspnea in patients with chronic interstitial lung disease, Faisal and colleagues observed that dyspnea intensity during exertion climbed as inspiratory neural drive increased and tidal volume became constrained (thus blunting the mechanical respiratory response during exercise) [15].

Another consideration is whether the greater disease severity in O_2_ users might have contributed to physical inactivity and deconditioning. Given the practical challenges of using O_2_ and the possibility that it prohibits patients from living a more active, carefree lifestyle, we suspect that, on average, fILD patients who require O_2_ are less physically active (and thus less well-conditioned) than patients who do not require O_2_. De-conditioned skeletal muscles are less efficient and fatigue-resistant than conditioned muscles, and peripheral locomotor [14] muscle fatigue contributes to dyspnea. Compared with non-users, O_2_ users walked shorter distances during the 6MWT; this was true even for the subgroup that did not desaturate to < 89 %. Whether the shorter distance walked was due to deconditioning or some other factor(s) is unknown; however, deconditioning could well explain the interesting and perhaps somewhat paradoxical finding that dyspnea severity was inversely correlated with 6MWD: subjects who walked further, were better conditioned and thus perceived less dyspnea.

## Conclusion

Dyspnea is a complex perception that impacts the lives of patients with fILD. It is important for a patient with fILD to know what to expect when being prescribed O_2_: it will likely decrease dyspnea (compared with not using O_2_), but because dyspnea is driven by so many inputs (with SpO_2_ being just one), O_2_ will not resolve dyspnea. Further research is needed to better understand the mechanisms driving dyspnea in patients with fILD and to devise strategies to lessen it.

## Abbreviations

6MWT: six-minute walk test
6MWD: distance walked during a six-minute walk test
cHP: chronic hypersensitivity pneumonia
COPD: chronic obstructive pulmonary disease
CTD: connective tissue disease
fILD: fibrosing interstitial lung disease
FVC%: percent predicted forced vital capacity
ILD: interstitial lung disease
iNSIP: idiopathic nonspecific interstitial pneumonia
IPF: idiopathic pulmonary fibrosis
IRB: institutional review board
O2: supplemental oxygen
QOL: quality of life
SpO2: peripheral oxygen saturation
